# Giant intrathoracic ganglioneuroma with scoliosis treated by one-stage posterior resection and scoliosis correction: a case report

**DOI:** 10.1051/sicotj/2020012

**Published:** 2020-05-07

**Authors:** Belal Elnady, Ahmed Shawky Abdelgawaad, Hussein Elkhayat

**Affiliations:** 1 Department of Orthopedic and Trauma Surgery, Assiut University Hospitals 71111 Assiut Egypt; 2 Department of Spine Surgery, Helios Klinikum Erfurt 99089 Erfurt Germany; 3 Department of Cardiothoracic surgery, Assiut University Hospitals 71111 Assiut Egypt

**Keywords:** Giant ganglioneuroma, Ganglioneuroma with scoliosis, Spinal ganglioneuroma, Posterior approach

## Abstract

Spinal ganglioneuroma occurs mostly in the thoracic spine causing various manifestations including scoliosis that can be misdiagnosed as idiopathic scoliosis. Few reports exist in the literature on the diagnosis and management of scoliosis secondary to huge ganglioneuroma and usually staged treatment is preferred. In this report, we present a 17-year-old female patient presented with back pain, lower limbs numbness, spinal deformity, and shortness of breath. Plain X-rays showed a 50° right thoracic scoliotic curve. MRI and chest CT revealed a huge extra pulmonary mass shifting the mediastinum with intra spinal extension through the left neural foramina compressing the spinal cord. Percutaneous US guided needle biopsy confirmed the diagnosis of ganglioneuroma. One-stage posterior instrumented correction of scoliosis, spinal cord decompression, and excision of the whole mass from the mediastinum and the spine through posterior approach was done for the patient with smooth postoperative recovery. Chest CT scan was done 2 years after surgery and excluded any local recurrence.

## Introduction

Ganglioneuroma (GN) is a slowly progressing benign neural tumor arising from sympathetic ganglion [1]. Spinal ganglioneuroma occurs most commonly in the thoracic spine [2] causing various manifestations including pain, radiculopathy, paraparesis, and scoliosis [3].

Few reports exist in the literature on the diagnosis and management of scoliosis secondary to giant ganglioneuroma and usually staged treatment is preferred [4].

In this report, we present a 17-year-old female patient with giant ganglioneuroma in the thoracic spine causing scoliosis with intra thoracic extension treated by one-stage posterior excision from both the spine and the chest together with instrumented correction of associated scoliosis.

To the best of our knowledge, this is the first report on one-stage posterior excision of giant mediastinal GN with intra spinal extension together with scoliosis correction.

## Case presentation

**Figure 1:**
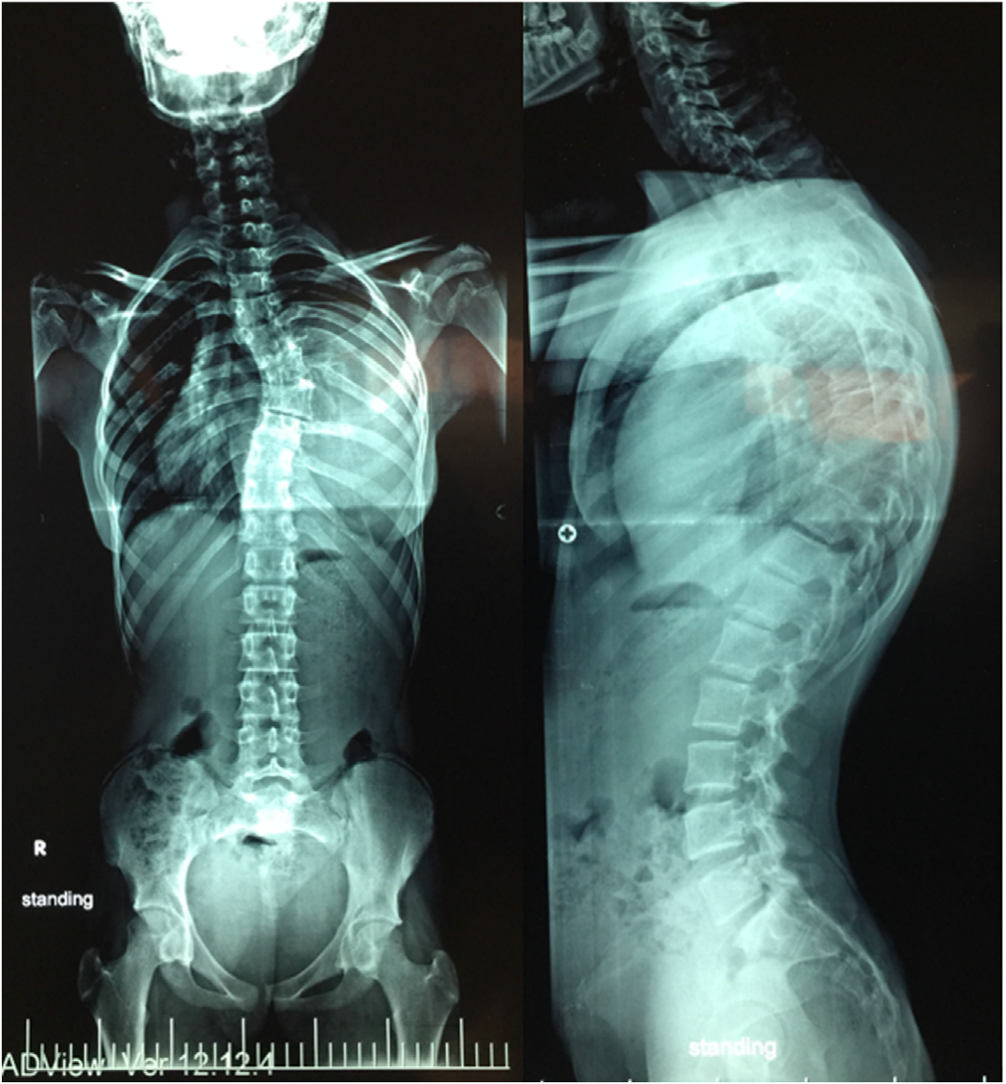
Preoperative full-length PA and lateral whole spine X-rays showing a 50° right thoracic scoliotic with thoracic kyphosis of 65°.

**Figure 2:**
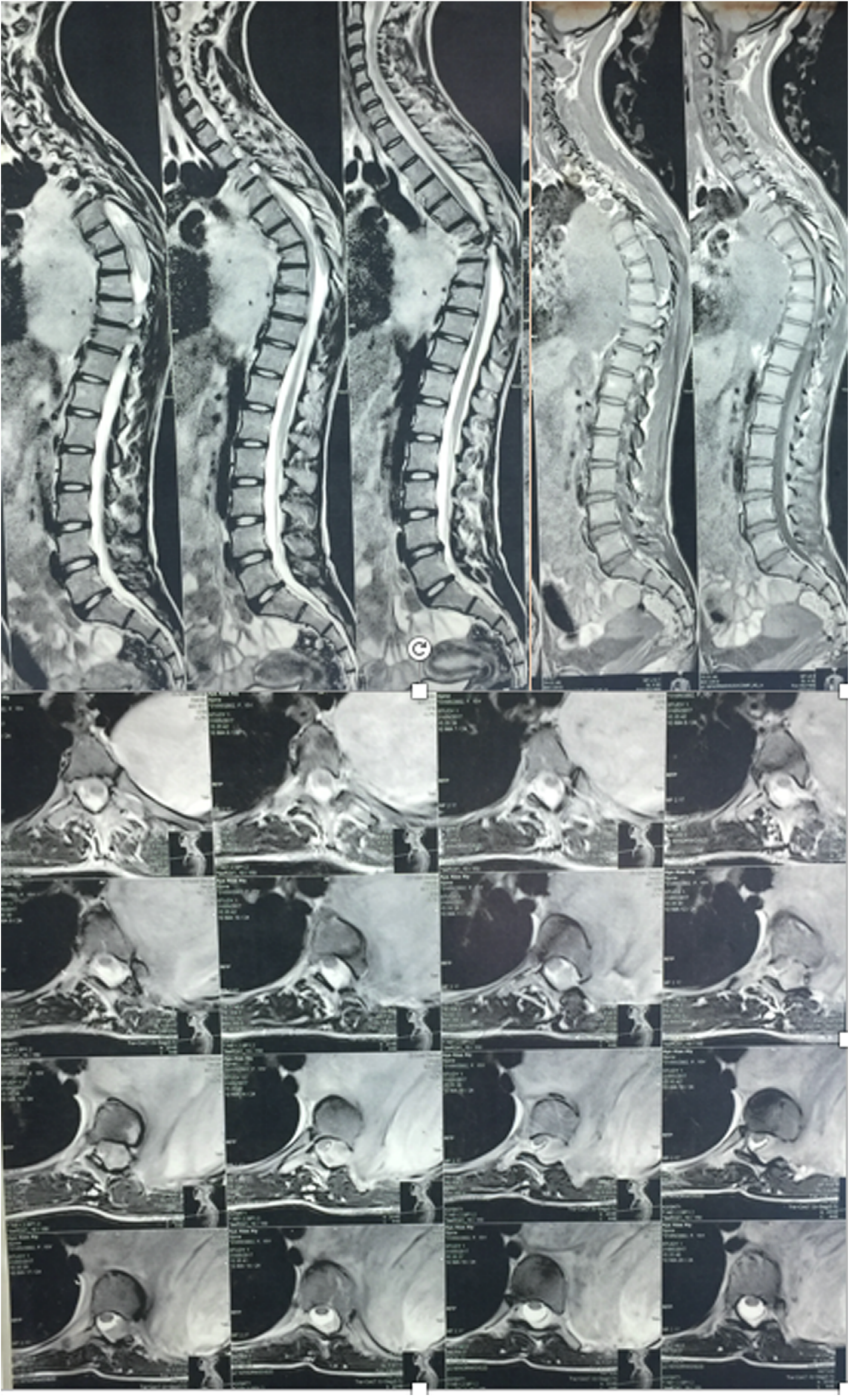
Preoperative MRI revealing a giant left extra pulmonary mass with intra spinal extension compressing the spinal cord.

**Figure 3:**
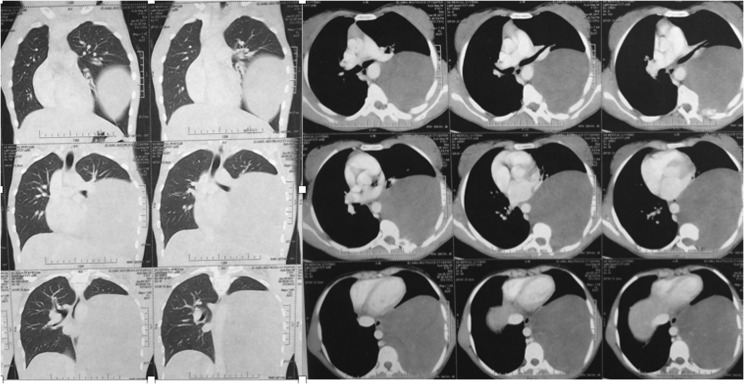
Preoperative chest CT scan showing the giant mass in the left hemithorax shifting the mediastinum to the right side.

A 17-year-old female patient was presented to our institute on April 2016 complaining of occasional back pain and spinal deformity. On examination, the patient had no limitation of physical activity, occasional pain, no neurological deficits with mild thoracic scoliosis. Reassurance of the patient and her family was done with conservative treatment. Six months later, the patient presented back with worse pain, numbness of both lower limbs, progressive deformity, and shortness of breath. MRI was ordered for her, and revealed a giant extra pulmonary mass shifting the mediastinum to the opposite side, extending opposite T6 to the lower border of T10 with intra spinal extension through the left neural foramina compressing the spinal cord. Full-length spinal PA and lateral X-rays showed left thoracic scoliotic curve extending from T5 to T9 with apex at T7 and measuring about 50° with Cobb method with thoracic kyphosis of 65° (Figures 1–3).

Percutaneous US-guided needle biopsy was done, and the histopathological report confirmed the diagnosis of ganglioneuroma. One-stage posterior instrumented correction of scoliosis, spinal cord decompression, and excision of the whole mass through posterior approach was planned for the patient after a multidisciplinary team approval in our institute comprising spine surgeon, anesthesiologist, and cardiothoracic surgeon.

The patient was operated under general anesthesia in prone position over a radiolucent table. Standard posterior approach to the thoracic spine was done exposing the posterior elements subperiosteally. Then pedicle screws were inserted using the free hand technique from T3 to T11 and their position was checked by intra operative radiograph. A rod was connected to the screws on the right side and then wide microscopic decompression of the spinal canal was done exposing the mass and the spinal cord. Careful dissection of the mass from the spinal cord was done and the nerve root connecting the mass to the cord was identified and ligated. Ponte osteotomy was done at the apex and then correction was done by rod derotation technique. The second rod was inserted, then Wake-up test was done, and after confirming neural integrity, posterior spinal fusion was done using local bone graft.

**Figure 4:**
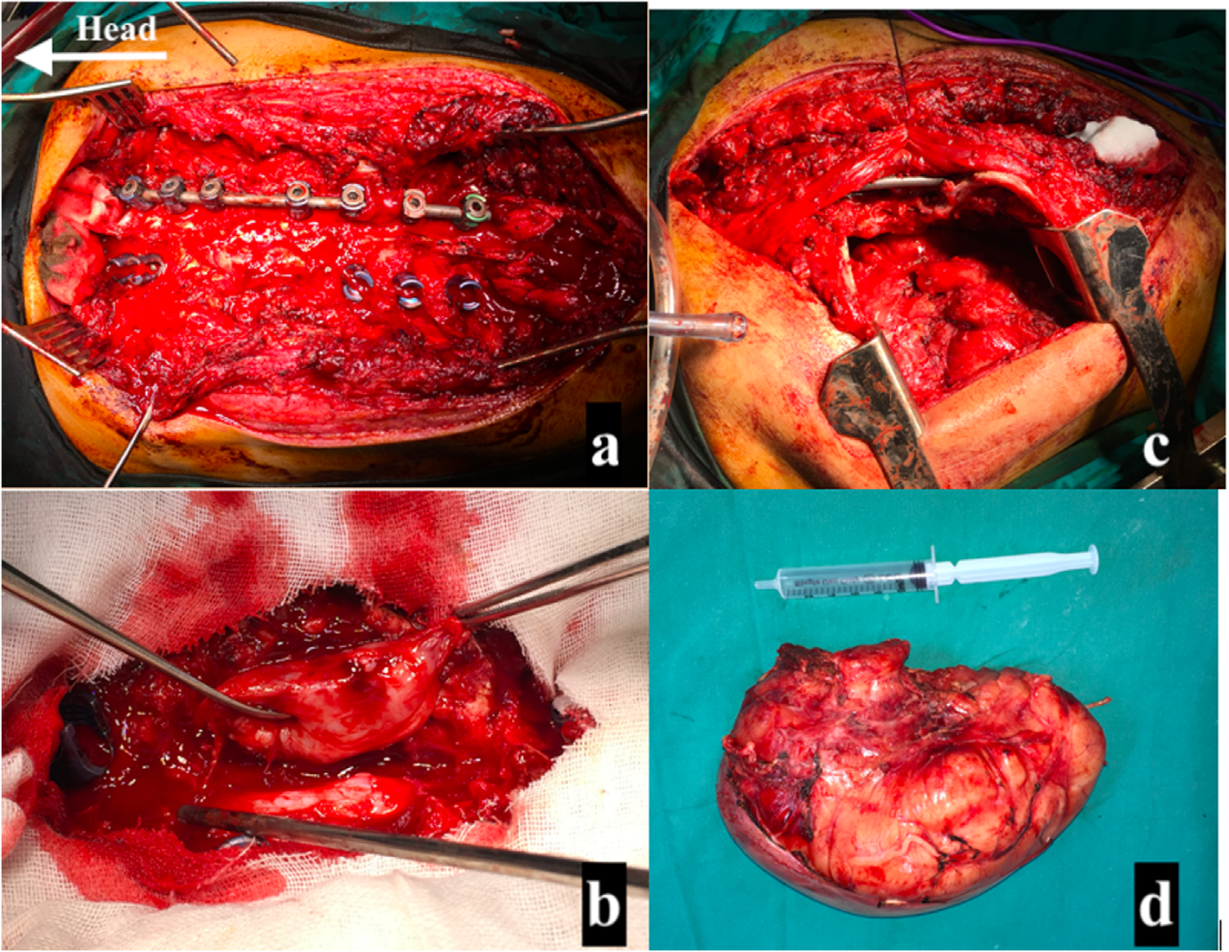
Intraoperative photos. (a) Pedicle screws insertion with rod connected to the right side and microscopic decompression of the spinal cord. (b) Separation of the mass from the cord and ligation of the connecting nerve root and excision of the mass from the spinal cord. (c) Retraction of the para-spinal muscles medially and entering the left hemithorax through the 6th intercostal space posteriorly. (d) The white tumor mass after complete resection.

After completion of the correction of scoliosis and excision of the intra-spinal extension on the tumor, thoracic surgeons approached the intrathoracic part of the tumor through the same incision by retraction of the back muscles medially and entering the left hemithorax through the 6th intercostal space posteriorly. Exploration of the chest showed no infiltration to the adjacent lung or mediastinal structure. Using an energy sealing device (Liga-Sure Impact™ sealer/divider, Medtronic, USA), the feeding branches from the descending aorta to the tumor were sealed and divided and then the tumor was removed en-bloc. Chest tube was inserted from a different stab in the anterior axillary line for drainage and ribs were approximated in the standard fashion; then back muscles were approximated and wound closed in layers (Figure 4).

**Figure 5:**
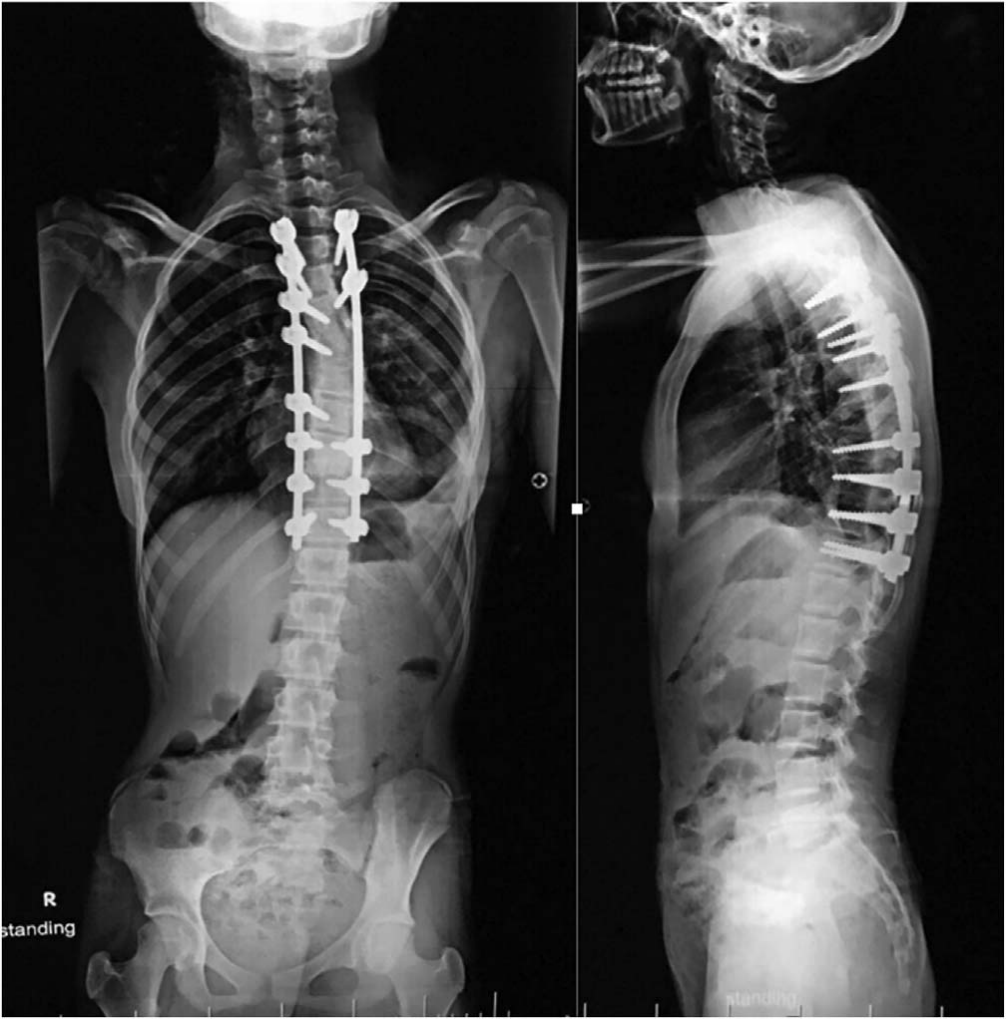
Postoperative full-length PA and lateral whole spine radiograph showing the correction of the scoliotic curve to 30° and the thoracic kyphosis to 32°.

Patient was successfully extubated on table and transferred to ICU where she was monitored for vital signs, chest tube output, and neurological manifestation and she was on IV opioids for the first 48 h. Mobilization was possible from the second day postoperative and chest tube was removed on the 4th day. Postoperative radiograph showed the correction of the scoliotic curve to 30° and the thoracic kyphosis to 32°. Patient was discharged home on the 6th day postoperative uneventfully (Figure 5).

The tumor was a firm white mass measuring about 20 × 15 × 8 cm with smooth outer surface. Serial cut sections showed homogenous soft to firm white cut surfaces. Microscopic examination revealed sweeping long and short fascicles with wavy spindle cells and numerous mature ganglion cells in-between surrounded with hyaline and myxoid stroms. The histopathological diagnosis was consistent with ganglioneuroma. The patient was followed up at 2 weeks, 2 months, 1 year, and 2 years postoperatively. Chest CT scan was done 2 years after surgery and excluded any local recurrence.

## Discussion

Spinal ganglioneuroma constitutes about 10% of all ganglioneuromas and most commonly occurring in the thoracic spine [2]. Spinal ganglioneuroma usually appears as a dumbbell-shaped mass involving the para-spinal region and extending into the spinal canal through the neural foramina causing various manifestations including radiculopathy, paraparesis, and scoliosis [4].

The mechanism by which GN can cause scoliosis is still not clear [5]. A misdiagnosis of scoliosis due to GN as idiopathic scoliosis is not uncommon. Careful evaluation of the patient and the curve before surgery is mandatory to avoid misdiagnosis. Patients with atypical curve patterns, pain, rapid progression, and neurological symptoms should be thoroughly investigated before a diagnosis of idiopathic scoliosis is made [5].

D’Eufemia et al. [4] reported a case of misdiagnosed GN as adolescent idiopathic scoliosis (AIS). He reviewed the literature and found nine articles dealing with GN and scoliosis. In seven of them, patients were misdiagnosed as AIS and treatment was delayed.

As GN can reach a huge size while asymptomatic due to slow progression, he identified red flags to suspect secondary scoliosis that needs MRI to avoid delay on treatment. Those red flags included back pain not responding to anti-inflammatory treatment, curve progression and stiffness, neurological deficits, and atypical curve patterns [4].

Yang et al. [6] reviewed the literature about the incidence of GN and scoliosis in the past 40 years; he found 16 cases in 13 papers and he recommended to divide surgical treatment into two stages with 1–7-week interval in-between. Qiu et al. [5] reported two patients with scoliosis secondary to GN treated also by two-stage surgery.

Wang et al. [7] described one-stage surgery for excision of lumbar GN with stabilization and fusion. However, no associated scoliosis or intra thoracic extension was in his case.

In this study, Single posterior approach was done to correct scoliosis, decompress the spinal cord, and excise the whole mass from the mediastinum and the spine. The posterior approach to the spine is simple, extensile, and familiar to all spine surgeons with little risk of vascular or visceral complications [8]. This saved our patient from combined approaches or staged surgeries and their morbidity [9, 10].

The aim of the surgery was to excise the tumor completely and stabilize the curve to prevent further progression after laminectomy with restoration of the thoracic kyphosis. The scoliosis curve was rigid and we accepted partial correction of the curve to avoid more aggressive corrective maneuvers like asymmetrical PSO that would increase the operative time, manipulation of the cord, and blood loss.

Using the same incision for approaching the intrathoracic part of the mass ensured that the mass was excised in continuity en-bloc with proper intercostal space entry to the chest as we started the incision from the medial aspect of the mass. Prone position also helped dissecting the feeding vessels arising from the aorta easily instead of making too much tension on the mass with anterior retraction in case of the conventional posterolateral thoracotomy. Same incision provides a very good cosmetic result added on to the correction of scoliosis and better patient overall postoperative pain experience.

Spinal GN had low recurrence rate with no need for postoperative chemotherapy or radiotherapy with good prognosis after complete resection [11]. A multidisciplinary team of chest surgeon, spine surgeon, and anesthesiologist improved the outcome and facilitated planning for a successful surgery.
